# Everyday ageism experienced by community-dwelling older people with frailty

**DOI:** 10.1007/s41999-024-01048-0

**Published:** 2024-09-08

**Authors:** Saidhbh Comerford, Ellie O’Kane, Domhnall Roe, Hamad Alsharedah, Benny O’Neill, Michael Walsh, Robert Briggs

**Affiliations:** 1 Mercer’s Institute for Successful Ageing, St James’s Hospital, Dublin, Ireland; 2 School of Medicine, Trinity College Dublin, Dublin, Ireland; 3 Discipline of Medical Gerontology, Trinity College Dublin, Dublin, Ireland

**Keywords:** Multimorbidity, Quality of life, Ageist, Discrimination

## Abstract

**Purpose:**

Ageism encapsulates stereotypes, prejudice and discrimination towards others or oneself based on age. While identified as the most frequent form of discrimination, relatively little work has captured the problem of day-to-day ageism faced by older people with frailty in community settings. The aim of this study therefore was to examine the prevalence of everyday ageism experienced by older people attending hospital-based ambulatory care services, and to clarify its association with measures of quality of life (QOL) and frailty.

**Methods:**

A consecutive series (n = 100) of patients aged ≥ 70 years attending ambulatory care completed focused interviews. Day-to-day experience of ageism was measured with the everyday ageism scale, QOL was measured with the control autonomy self-realisation pleasure scale.

**Results:**

Just over half of participants reported exposure to ageist messages; over 5/6 reported experiencing ageism in interpersonal interactions and 2/3 held some ageist beliefs themselves. Logistic regression demonstrated that experiencing ageism in interpersonal interactions was associated with a higher likelihood of internalised ageism [Adjusted Odds Ratio 6.02 (95% CI 1.70, 21.34); p = 0.005)]. Both ageism in interpersonal interactions [β = −5.22 (95% CI −9.52, −0.91); p = 0.018] and internalised ageism [β = −5.36 (95% CI −8.75, −1.97); p = 0.002] were associated with significantly lower QOL.

**Discussion:**

This study highlights the striking prevalence of everyday ageism experienced by a cohort of community-dwelling older people with frailty and multimorbidity.

In the context of further projected demographic changes in coming decades, with increasingly higher proportions of older people worldwide, these findings highlight an important societal issue that needs to be addressed.

**Graphical abstract:**

## Key summary points

**Aim:**

To examine the prevalence of everyday ageism experienced by older people attending hospital-based ambulatory care services, and to clarify its association with measures of quality of Life (QOL).

**Findings:**

Just over half of participants reported exposure to ageist messages; over 5/6 reported experiencing ageism in interpersonal interactions and 2/3 held some ageist beliefs themselves. Both ageism in interpersonal interactions and internalised ageism were associated with significantly lower QOL.

**Message:**

In the context of further projected demographic changes in coming decades, with increasingly higher proportions of older people worldwide, these findings highlight an important societal issue that needs to be addressed.

## Graphical abstract

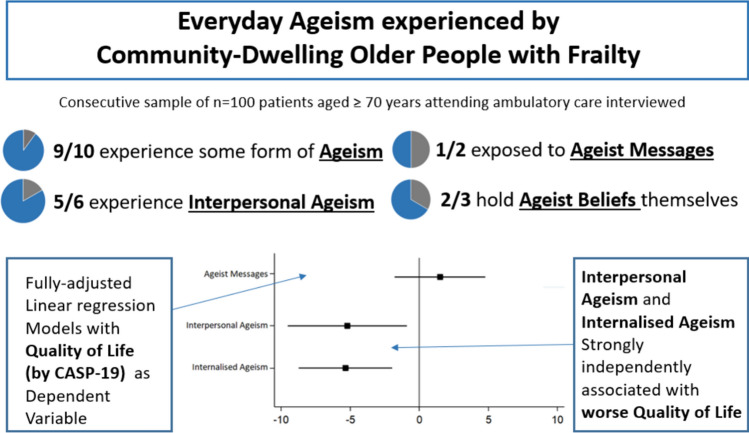

## Background

Living into old age offers opportunities, both for the individual and their families, but also for society in general, and is something most people rightly aspire to. This ‘longevity dividend’, facilitates productive contribution from older people in areas such as employment and the economy [1], community and social engagement and participation [2], care giving [3] and creativity [4]. It is troubling then that the process of getting older can frequently be viewed pessimistically by both younger and older people [5, 6].

Ageism encapsulates the stereotypes (how we think), prejudice (how we feel) and discrimination (how we act) towards others or oneself based on age [7] and is the most frequent form of discrimination [8]. While ageism can affect people at all stages of life, later life is the most frequent time ageism is experienced and when it is most harmful [9]. Internalised or self-directed ageism refers to ageism turned against oneself [8].

Ageism is as an important determinant of health, with the recent World Health Organisation global report on ageism [7] outlining the detrimental effect it can have on well-being and life expectancy, across geography and time. Further, ageism has a negative impact at an organisational level, leading to denied access to healthcare and treatments, exclusion from clinical trials, devalued lives and lack of work opportunities [10].

The impact of ageism was brought into focus during the COVID-19 pandemic [11], demonstrated by the lack of preparation for outbreaks in nursing homes, discussions around prioritising younger patients for intensive care beds, how older people were directed to cocoon without due concern for the physical and psychological impact of these measures [12] and how the deaths of older people were documented during the pandemic [13].

While several studies have explored ageism in terms of major events like the COVID-19 pandemic, healthcare access [14] or inclusion in research studies [15], relatively little work to date has captured the problem of day-to-day ageism faced by older people with frailty in community settings.

The aim of this study therefore was to examine the prevalence of everyday ageism experienced by community-dwelling older people attending hospital-based ambulatory care services, and to clarify its association with measures of well-being and frailty. Furthermore, we also assessed if these patients held ageist attitudes or beliefs themselves, and the association between such internalised ageing and exposure to external everyday ageism.

## Methods

### Study design

This cross-sectional study ascertained the prevalence of everyday ageism, and its association with frailty and quality of life, in a cohort of outpatients aged ≥ 70 years attending specialist ambulatory care services for older people in a tertiary referral unit for geriatric medicine.

A consecutive series (n = 100) of patients aged ≥ 70 years attending ambulatory care (outpatient clinic for older people or day hospital) in March 2023 were included. Patients were not eligible for inclusion if they could not give informed consent or were unable or unwilling to complete the interview.

### Everyday ageism

Day-to-day experience of ageism was measured with the everyday ageism scale, a multidimensional 10-item Likert scale, scored from 0–30, with higher scores indicating higher levels of experienced ageism [16].

The scale has a three-factor structure including exposure to ageist messages in the form of environmental and social cues (items 1–2); ageism in interpersonal interactions, specifically being subject to discrimination based on assumptions about older adults (items 3–7); and endorsement of internalized ageism, reflecting individually held beliefs linking aging and health (items 8–10) [16]. Examples of items include ‘I hear, see and/or read jokes about old age, ageing or older people’ (ageist messages) ‘People assume I have difficulty remembering and/or understanding things’ (ageism in interpersonal interactions) and ‘Feeling lonely is part of getting older’ (internalised ageism), with participants asked to respond with often (3), sometimes (2), rarely (1), or never (0) [16].

For each of these three subscales, if a respondent answered rarely or sometimes to any relevant items, they were considered to experience that aspect of ageism, i.e. exposure to ageist messages, ageism in interpersonal interactions and/or internalised ageism.

### Quality of life

Quality of life was measured with the control autonomy self-realisation pleasure scale (CASP-19), which was developed for use and validated in older people [17]. CASP-19 is a likert scale involving four domains (control, autonomy, self-realisation, and pleasure) to assess the quality of life scored from 0 to 57, with higher scores indicating a better quality of life. Examples of items include ‘I feel that what happens to me is out of my control’ and ‘I feel that life is full of opportunities’, with participants asked to respond often, sometimes, not often or never.

### Frailty

Frailty, a syndrome characterised by increased vulnerability to stressors, with higher risk of adverse health outcomes [18], was measured with the clinical frailty scale (CFS) [19].

The CFS is an inclusive 9-point scale that uses clinical judgement to summarise the baseline level of fitness or frailty of an older person [19]. It focuses on items that can be readily observed, including mobility, and the abilities to eat, get dressed, do shopping and cook [20]. Training was provided on CFS assessment. The CFS was completed by physicians or trainee physicians with experience in Geriatric Medicine and cross-checked by a Consultant Geriatrician.

### Other measures

Further data collected included:

- Basic demographics (age, sex)
- Living arrangements (alone, with partner or with others)
- Number of chronic illnesses (as documented in medical notes)

### Statistical analysis

Data was presented descriptively and analysed using Stata 14 (Statacorp). Binary variables were presented as proportions with 95% confidence intervals. Differences in mean values were analysed with t-tests and t-statistic and p value were reported.

Linear regression models, reporting β co-efficients with 95% confidence intervals and with CASP-19 score as the dependant variable, were used to assess the relationship between ageism (ageist messages, interpersonal interactions and internalised ageism) and quality of life. Analysis was adjusted for age (in categories 70–75 years, 76–80 years, 80–85 years and ≥ 86 years), sex, living arrangements (alone, with partner, with family/others), CFS (in categories CFS 1–3, CFS 4, CFS 5, CFS 6–7) and number of medical comorbidities (in categories < 3, 4–5, 6). Additional two-way interaction models were used to assess the relationship between interpersonal and internalised ageism in terms of the association with quality of life.

Logistic regression models, reporting odds ratios with 95% confidence intervals, were used to examine the association between ageism and binary variables of interest.

## Results

### Baseline characteristics

Mean age of participants (n = 100) was 80.4 (95% CI 79.2–81.7) years. One fifth of participants [proportion 0.20 (95% CI 0.13–0.29)] were aged over 85 years. Over half [proportion 0.56 (95% CI 0.46–0.66)] were female. Two in five were living alone [proportion 0.41 (95% CI 0.32–0.51)].

Mean CFS was 4.7 (95% CI 4.5–5.0), with over one third of participants [proportion 0.34 (95% CI 0.25–0.44)] with a CFS ≥ 6. Mean number of medical comorbidities was 4.98 (95% CI 4.51–5.45).

Almost nine in 10 participants reported exposure to either ageist messages or ageism in interpersonal interactions [proportion 0.89 (95% CI 0.81–0.94)].

Mean score on the everyday ageism scale was 12.52 (95% CI 11.38–13.67).

### Exposure to ageist messages

Just over half of participants reported exposure to ageist messages [proportion 0.51 (95% CI 0.41–0.61].

This includes hearing, seeing and/or reading jokes about older age, ageing or older people [proportion 0.39 (95% CI 0.30–0.49)] and hearing, seeing and/or reading things suggesting that older adults and aging are unattractive [proportion 0.31 (95% CI 0.23–0.41)].

There was no age difference between those reporting exposure [mean age 81.0 (95% CI 79.2–82.9) years] and those who denied exposure [mean age 79.8 (95% CI 78.1–81.5) years] to ageist messages (t = −0.9609; p = 0.3390).

### Ageism in interpersonal interactions

Over 5 out of 6 participants reported experiencing ageism in interpersonal interactions [proportion 0.84 (95% CI 0.75–0.90)].

This includes reporting that people insist on helping them even if they can do things on their own [proportion 0.57 (95% CI 0.47–0.66)]; people assuming they have difficulty hearing and/or seeing things [proportion 0.43 (95% CI 0.34–0.53)]; people assuming they have difficulty remembering and/or understanding things [proportion 0.45 (95% CI 0.35–0.55)]; people assuming they have difficulty with mobile phones and computers [proportion 0.58 (95% CI 0.48–0.67)] and people assuming they do not do anything important or valuable [proportion 0.23 (95% CI 0.16–0.32)].

Participants who experienced ageism in interpersonal interactions were not significantly older [mean age 80.8 (95% CI 79.5–82.1)] than those who did not report interpersonal ageism [mean age 78.6 (95% CI 74.9–82.3)].

### Internalised ageism

Over two thirds of participants [proportion 0.70 (95% CI 0.60–0.78)] held some ageist beliefs themselves.

Over half of participants [proportion 0.53 (95% 0.43–0.63)] reported that they believed feeling depressed, sad, or worried is part of getting older; one third [proportion 0.32 (95% CI 0.23–0.42)] believed feeling lonely is part of getting older, while almost two thirds [proportion 0.60 (95% CI 0.50–0.69)] believed having health problems is part of getting older.

There was no significant age difference between those with and without internalised ageism [age 80.3 (95% CI 78.8–81.8) years in those with, 80.7 (95% CI 78.4–83.1) years in those without; t = 0.3159; p = 0.7528].

Participants reporting ageist beliefs were more likely to experience ageism in their interpersonal interactions [proportion 0.91 (95% CI 0.82–0.96) vs 0.67 (95% CI 0.47–0.82); X^2^ = 9.58; p = 0.002], but not more likely to be exposed to ageist messages [proportion 0.53 (95% CI 0.41–0.64) vs 0.47 (95% CI 0.29–0.65); X^2^ = 0.32; p = 0.570].

Logistic regression demonstrated that experiencing ageism in interpersonal interactions was significantly associated with a higher likelihood of internalised ageism in fully-adjusted models [adjusted odds ratio 6.02 (95% CI 1.70–21.34); p = 0.005)].

### Everyday ageism and quality of life

**Fig. 1:**
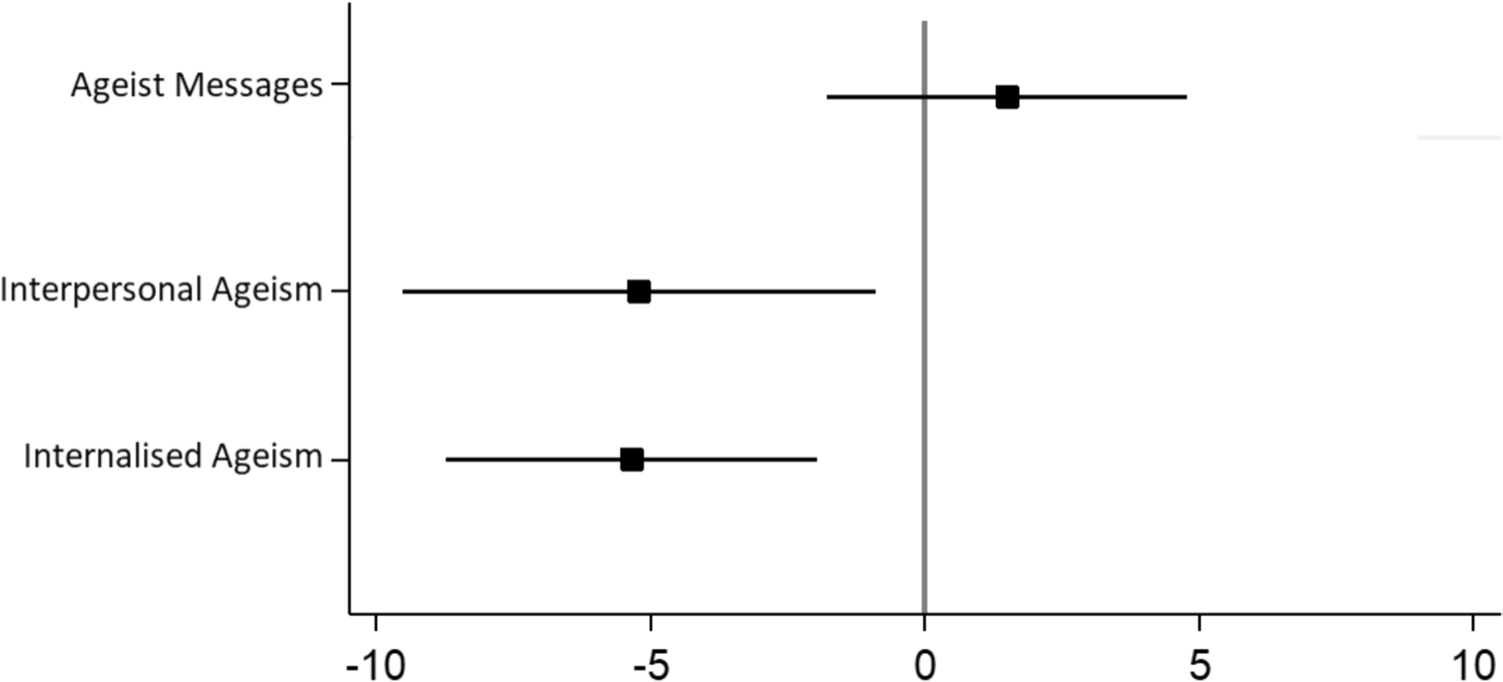
Fully-adjusted linear regression models with CASP-19 as dependent variable. Output from linear regression models, reporting beta-coefficients with 95% confidence intervals, with total score on CASP-19 as dependent variable. Analysis adjusted for age, sex, living arrangements (alone, with partner or with others), Clinical Frailty Score and number of chronic illnesses. Higher score on CASP-19 = better quality of life. Ageist messages, interpersonal ageism and internalised ageism were assessed separately, i.e. they were not included together in the model

As shown in Fig. 1, both ageism in interpersonal interactions [β = −5.22 (95% CI −9.52 to−0.91); p = 0.018] and internalised ageism [β = −5.36 (95% CI −8.75 to −1.97); p = 0.002)] were associated with significantly lower self-reported quality of life (measured by CASP-19), after adjusting for age, sex, living arrangements, frailty and burden of chronic disease. Exposure to ageist messages was not associated with quality of life in fully-adjusted models however [β = 1.48 (−1.79 to −4.76); p = 0.370].

When examined by CASP-19 subscales, ageism in interpersonal interactions was most strongly independently associated with lower scores for pleasure [β = −1.89 (95% CI −3.39 to −0.38); p = 0.014], followed by self-realisation (β = −1.50 (95% CI −3.03 to 0.04); p = 0.056), autonomy [β = −1.36 (95% CI −2.97 to 0.25); p = 0.096] and control [β = −0.47 (95% CI −1.61 to 0.67); p = 0.417]. Internalised ageism was most strongly associated with autonomy [β = −2.40 (95% CI −3.61 to −1.18); p < 0.001] and pleasure [β = −1.43 (95% CI −2.64 to −0.22); p = 0.021], followed by self-realisation [β = −0.88 (95% CI −2.13 to 0.36); p = 0.162] and control] β = −0.64 (95% CI −1.56 to 0.27); p = 0.163].

Two-way interaction models involving interpersonal and internalised ageism, demonstrated that the co-existence of these two types of ageism were more strongly associated with quality of life than either form in isolation. See Table 1.

**Table 1:** Two-way interaction linear regression models with CASP-19 as dependant variable

Dependant variable: CASP-19 score	Β-coefficient with 95% confidence interval	p
2-way interaction:		
No interpersonal or internalised ageism	Reference value	
Interpersonal ageism, no internalised ageism	−1.83 (−8.09, 4.42)	0.561
Internalised ageism, no interpersonal ageism	−3.08 (−11.16, 5.00)	0.451
Both internalised and interpersonal Ageism	−7.33 (−12.70, −1.97)	0.008
Age category (years):		
70–75	Reference value	
76–80	−2.44 (−6.92, 2.03)	0.280
81–85	−0.15 (−4.66, 4.37)	0.948
≥ 86	− 0.44 (−6.32, 5.43)	0.881
Female sex	−1.84 (−5.13, 1.46)	0.271
Living arrangement:		
Alone	Reference value	
With partner	−0.08 (−3.72, 3.57)	0.967
With another relative/friend	1.33 (−2.99, 5.66)	0.541
Clinical frailty scale:		
CFS 1–3	Reference value	
CFS 4	−2.69 (−7.49, 2.10)	0.268
CFS 5	−0.82 (−5.65, 4.00)	0.736
CFS 6–7	−3.22 (−8.17, 1.72)	0.199
Number of comorbidities:		
< 3	Reference value	
4–5	−1.78 (−5.68, 2.12)	0.366
≥ 6	−2.21 (−6.25, 1.84)	0.282

*CASP-19* 19-item control, autonomy, self-realisation pleasure scale, *CFS* clinical frailty scale

Ageism assessed with everyday ageism scale

### Everyday ageism and frailty

There was no difference in Mean CFS scores between those exposed [mean CFS 4.61 (95% CI 4.20–5.02)] and not exposed [mean CFS 4.88 (95% CI 4.52–5.24)] to ageist messaging (t = 0.99; p = 0.3242).

Participants who experienced ageism in interpersonal interactions [mean CFS 4.88 (95% CI 4.59–5.17)] had higher frailty scores than those who did not [mean CFS 4.00 (95% CI 3.27–4.73)] (t = −2.43; p = 0.0168).

Similarly, participants with internalised ageism [mean CFS 4.90 (95% CI 4.60–5.20)] had higher levels of frailty those who did not report ageist attitudes [mean CFS 4.33 (95% CI 3.80–4.86)] (t = −1.9857; p = 0.0499).

**Fig. 2:**
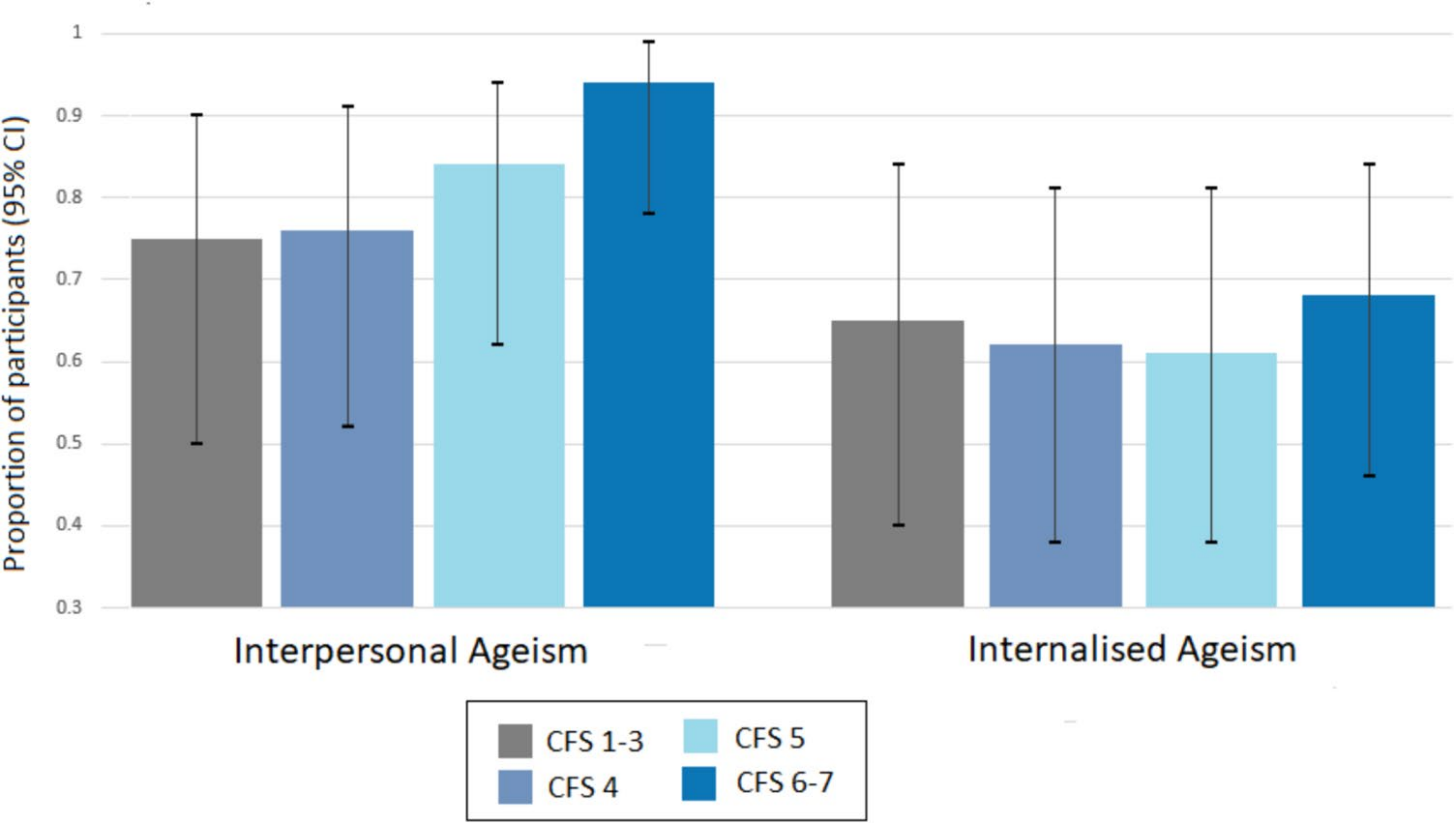
Interpersonal and internalised ageism by frailty. *CI* confidence interval, *CFS* clinical frailty scale. Everyday ageism scale used to assess interpersonal ageism (items 3–7) and internalised ageism (items 8–10). Clinical frailty scale used to assess frailty

The proportions of participants reporting ageism in interpersonal interactions and internalised ageism by CFS categories are shown in Fig. 2.

## Discussion

This study reports experience of everyday ageism in a cohort of older people attending ambulatory care services in a geriatric medicine unit.

We found that ageism was strikingly prevalent. Half of participants reported exposure to ageist messaging, and five out of six experience ageism in interpersonal interactions. While some of the examples of interpersonal ageism may be considered benign or non-malicious, for example helping an older person with something they could do on their own, almost one quarter of participants reported that other people assume they do not do anything of importance or value.

Conversely, we also found that over two thirds of participants had some ageist beliefs or attitudes themselves, most frequently reporting that health problems were an inevitable part of getting older. Participants with internalised ageism were over 6 times more likely to experience ageism in their everyday interpersonal interactions. Such internalised ageism has been shown to be particularly associated with poorer health outcomes [21].

While prevalence varies by setting and population, prior studies have also shown a generally high prevalence of experienced ageism [22]. In a large European study, over one third of participants aged ≥ 65 years experienced unfair treatment because of their age [23], while almost half of a nationally representative sample of US adults ≥ 50 years reported experiencing interpersonal ageism [21]. Further, a large analysis of over 84,000 participants across 57 countries demonstrated that at least one in two had moderate or high ageist attitudes [24]. To our knowledge this is the first study to examine everyday ageism amongst a cohort of older patients in a hospital-setting, and assess its relationship with frailty and quality of life.

Both ageism in interpersonal interactions and internalised ageism were strongly independently associated with quality of life, measured by CASP-19, particularly the autonomy and pleasure domains and less so the control domain. Prior studies have consistently shown that internalised ageism especially is closely linked with quality of life amongst older people [25, 26]. When further assessed with 2-way interaction models in this study however, the co-existence of both internalised and interpersonal ageism was more strongly associated with poorer quality of life than either form of ageism in isolation. Additionally, ageism was more closely associated with quality of life than markers of health such as frailty and chronic disease burden.

There are some limitations to this study that should be noted. As this study involves a relatively small cohort with a mean age of over 80 years and high levels of frailty and comorbidity, findings are not generalizable to community-dwelling older people. Further, the fact that this cohort is attending tertiary care for medical treatment may impact on their response to items such as the inevitability of health problems in later life or on quality of life scores. However, it is important that issues such as ageism are examined amongst older people with frailty, as they are typically underrepresented in population-based studies and research in general. Further, we robustly adjusted for both frailty and the burden of chronic illnesses in our models examining the link between ageism and quality of life.

In conclusion, this study highlights the striking prevalence of everyday ageism experienced by a cohort of community-dwelling older people with frailty and multimorbidity. Almost 9 in 10 experience either ageist messaging or ageism in interpersonal interactions. Ageism was strongly associated with poorer quality of life and a significantly higher likelihood of internalisation of ageist beliefs and attitudes. In the context of further projected demographic changes in coming decades, with increasingly higher proportions of older people, these findings highlight an important societal issue that needs to be addressed. However, studies show that ageism directed towards older people can be ameliorated with better education around ageing and positive intergenerational contact [27]. Further, increases in healthy life expectancy and in the proportion of older people in a country are also associated with a lower likelihood of an individual holding ageist beliefs, while the quality of intergenerational contact and how ageing is presented are also important factors [24, 28]. The increased longevity we are now experiencing, and will continue to experience [29], can therefore be seen as an important opportunity to transform people’s expectations and experiences of ageing, as well as intergenerational relationships.
